# The impact of a prescription review and prescriber feedback system on prescribing practices in primary care clinics: a cluster randomised trial

**DOI:** 10.1186/s12875-018-0808-4

**Published:** 2018-07-19

**Authors:** Wei Yin Lim, Amar Singh HSS, Li Meng Ng, Selva Rani John Jasudass, Sondi Sararaks, Paranthaman Vengadasalam, Lina Hashim, Ranjit Kaur Praim Singh

**Affiliations:** 1 Clinical Research Centre Perak, Ministry of Health Malaysia, Level 4, Ambulatory Care Centre, Raja Permaisuri Bainun Hospital, Jalan Raja Ashman Shah, 30450 Ipoh, Perak Malaysia; 2 Department of Paediatrics, Raja Permaisuri Bainun Hospital, Ministry of Health Malaysia, Jalan Raja Ashman Shah, 30450 Ipoh, Perak Malaysia; 3 Manjung Health District Office, Ministry of Health Malaysia, Jalan Dato’ Ahmad Yunus, 32000 Sitiawan, Perak Malaysia; 4 Sg Chua Health Clinic, Ministry of Health Malaysia, Kaw Perindustrian Sg Chua, Sg Ramal Luar, 43000 Kajang, Selangor Malaysia; 5 Institute for Health Systems Research, Ministry of Health Malaysia, No. 2 Jalan Setia Prima S U13/S, Seksyen U13 Setia Alam, ,40170 Shah Alam, Selangor Malaysia; 6 Jelapang Health Clinic, Ministry of Health Malaysia, 30020 Ipoh, Perak Malaysia; 7 Perak State Health Department, Ministry of Health Malaysia, Jalan Panglima Bukit Gantang Wahab, 30590 Ipoh, Perak Malaysia

**Keywords:** Prescribing errors, P-chart, Statistical process control chart, League tables, Feedback, Prescription review, Primary care, Prescribers

## Abstract

**Background:**

To evaluate the effectiveness of a structured prescription review and prescriber feedback program in reducing prescribing errors in government primary care clinics within an administrative region in Malaysia.

**Methods:**

This was a three group, pragmatic, cluster randomised trial. In phase 1, we randomised 51 clinics to a full intervention group (prescription review and league tables plus authorised feedback letter), a partial intervention group (prescription review and league tables), and a control group (prescription review only). Prescribers in these clinics were the target of our intervention. Prescription reviews were performed by pharmacists; 20 handwritten prescriptions per prescriber were consecutively screened on a random day each month, and errors identified were recorded in a standardised data collection form. Prescribing performance feedback was conducted at the completion of each prescription review cycle. League tables benchmark prescribing errors across clinics and individual prescribers, while the authorised feedback letter detailed prescribing performance based on a rating scale. In phase 2, all clinics received the full intervention. Pharmacists were trained on data collection, and all data were audited by researchers as an implementation fidelity strategy. The primary outcome, percentage of prescriptions with at least one error, was displayed in p-charts to enable group comparison.

**Results:**

A total of 32,200 prescriptions were reviewed. In the full intervention group, error reduction occurred gradually and was sustained throughout the 8-month study period. The process mean error rate of 40.7% (95% CI 27.4, 29.5%) in phase 1 reduced to 28.4% (95% CI 27.4, 29.5%) in phase 2. In the partial intervention group, error reduction was not well sustained and showed a seasonal pattern with larger process variability. The phase 1 error rate averaging 57.9% (95% CI 56.5, 59.3%) reduced to 44.8% (95% CI 43.3, 46.4%) in phase 2. There was no evidence of improvement in the control group, with phase 1 and phase 2 error rates averaging 41.1% (95% CI 39.6, 42.6%) and 39.3% (95% CI 37.8, 40.9%) respectively.

**Conclusions:**

The rate of prescribing errors in primary care settings is high, and routine prescriber feedback comprising league tables and a feedback letter can effectively reduce prescribing errors.

**Trial registration:**

National Medical Research Register: NMRR-12-108-11,289 (5th March 2012).

**Electronic supplementary material:**

The online version of this article (10.1186/s12875-018-0808-4) contains supplementary material, which is available to authorized users.

## Background

Adverse drug events (ADEs) are one of the important causes of morbidity and mortality in primary care [1–3]. Such adverse events are frequently associated with medication errors [4–6], which can occur during any step of the medication use process–prescribing, transcribing, dispensing, administration, and monitoring [7]. Prescribing, the first stage in the medication use pathway, is most often the source of a series of drug-related problems in the healthcare system. A systematic review in 2007 reported that the largest proportion of errors in ambulatory care originated in the prescribing stage, accounting for 64.7% of all preventable ADEs, and 56.0% of preventable ADEs resulting in hospital admission [8].

Medication safety in primary care is considerably important, as primary care clinics are increasingly responsible for complex medication regimens, and a large portion of medical encounters are with government primary care practices [9]. However, despite primary care services playing an integral part of the healthcare delivery, quality improvement programs are less widespread in ambulatory settings compared to inpatient settings. Given that prescribing errors are theoretically preventable, interventions should target errors in prescribing [8].

Several published articles have described programs designed to improve physician prescribing behaviour. Recent reviews suggest that, in order to change prescribing behaviour, an active intervention is required [10–14]. Implementation research has also revealed the ineffectiveness of passive strategies such as printed education materials on actual prescribing behaviour, while relatively more active strategies such as audit with on-going feedback have shown greater promise in certain types of prescribing practices [11, 15]. However, the positive impact of audit with feedback was inconclusive [16, 17]. A Cochrane systematic review in 2006 [18] and the updated review in 2012 [19] concluded that the impact of audit and feedback on professional practice is small but potentially important, and is affected by the feedback delivery mechanism.

There is relatively little research about improving prescribing practice in primary care. As providing feedback is proven useful for changing behaviour, we aimed to design a prescription review and prescriber feedback program and to evaluate its effectiveness on improving prescribing practices in government primary care clinics. We designed the study to minimally change daily practices as the ultimate aim was to improve prescribing quality in the primary care system nationwide.

## Methods

### Study design and participants

We conducted a three-group pragmatic cluster randomised controlled trial among government primary care clinics with pharmacists in Perak, Malaysia. We chose a cluster design because the intervention was applied at the health clinic level. Of a total 79 government health clinics in Perak providing primary care services, 55 clinics with pharmacists were identified for the study, as the intervention involved prescription review by pharmacists. Excluded were health clinics with electronic prescribing systems, as the quality of computerised prescribing differed from that of manual prescribing [20]. Primary care prescribers of the remaining 51 health clinics were the target of this pragmatic intervention. We included prescribers of different expertise levels: family medicine specialists, medical officers, and medical assistants, all who routinely prescribe medications to patients in the primary care setting. Handwritten prescriptions of these prescribers were prospectively reviewed by pharmacists to screen for prescribing errors.

**Fig. 1:**
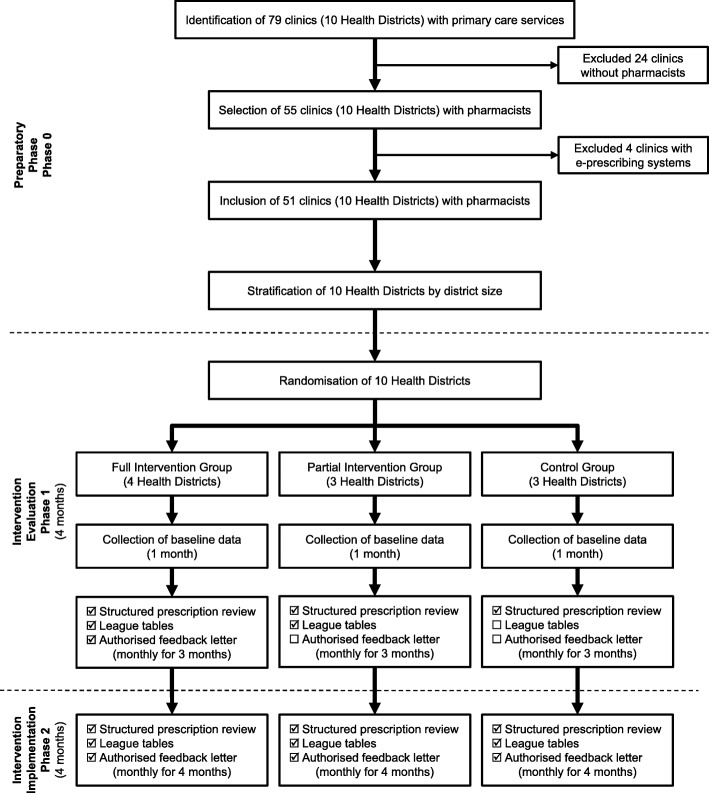
Summary of trial methodology flow chart

This pragmatic trial was conducted over eight months in two phases (May to December 2012). In phase 1 (May to August 2012), health clinics were randomised into one of three groups to receive either a) full feedback intervention [structured prescription review and prescribing performance feedback (league tables and authorised feedback letter)], b) partial feedback intervention [structured prescription review and prescribing performance feedback (league tables only)], or c) usual care as control (structured prescription review only). Prescription data collected in May 2012 represented the baseline data for the study. Interventions based on group allocation were implemented after the collection of baseline data. At the end of this phase, the prescribing performance of these three groups were compared to determine the intervention’s effect on error rates. In phase 2 (September to December 2012), all study groups received full feedback intervention, and prescribing performance was monitored until the end of the phase. In December 2012, the last batch of prescribing performance feedback was sent to prescribers and the trial ended. See Fig. 1 for the trial methodology flow chart of the trial.

### Allocation and blinding

In Perak, the 51 eligible health clinics come under the administration purview of ten health districts, each headed by a district health officer. Therefore, for the purpose of implementing the intervention program, we stratified the ten health districts by size (large: ≥6 health clinics, small: < 6 health clinics) and randomly allocated them in a 1:1:1 ratio to three parallel groups: full intervention group (full feedback intervention), partial intervention group (partial feedback intervention), and control group (usual care) by simple randomisation. Stratification by district size was to obtain an even distribution of large and small districts in each study group.

Due to the nature of the community interventions, it was not possible to blind neither the pharmacists nor the prescribers. The prescribers in the full and partial intervention groups were aware of the interventions when the first batch of prescribing performance feedback was mailed to them after the collection of baseline data. It was also not possible to blind the data analyst because the intervention program was implemented continuously and monthly feedback was provided to prescribers. However, the intervention assignments were concealed to the pharmacists and prescribers prior to randomisation.

### Prescription review and prescriber feedback program

As a pragmatic trial, state managers and clinicians were engaged throughout the study design, especially with designing the intervention program and its implementation work flow. The intervention program consists of two core components: a) structured prescription review and b) prescribing performance feedback containing two items: i) league tables and ii) authorised feedback letter. The structured prescription review component is built upon the basic duty of pharmacists—review prescriptions presented to them and intervene when necessary to ensure that prescriptions are therapeutically appropriate for patients. As this component closely resembles the pharmacist’s daily professional practice, it is therefore considered as ‘usual care’, albeit more structured and standardised (see Structured prescription review by pharmacists and Training of pharmacists).

#### Structured prescription review by pharmacists

Structured prescription review was conducted monthly by pharmacists in the health clinics. This process was conducted in accordance with a structured protocol prepared by the researchers; pharmacists were specially trained to conduct comprehensive prescription reviews (see Training of pharmacists), and researchers regularly audited this process to ensure its quality throughout the study. Handwritten prescriptions of prescribers in eligible clinics were consecutively collected by pharmacists and screened for errors. Pharmacists were required to collect 20 prescriptions per prescriber for up to five working days, starting from a randomly selected date in each month. To ensure that all prescription reviews were consistently conducted across health clinics, we constructed a standardised form for data collection. Errors identified in the prescriptions were recorded in the Prescribing Error Record Form (see Additional file 1). The definition of a prescribing error was adapted from that of Dean et al. [21]. The prescriptions were checked for: incomplete patient and prescriber information, incomplete dosing regimen (duration/quantity, dose, frequency, dosage form, strength), illegibility, use of non-standard abbreviations, inappropriate/incorrect dosing regimen (dose, frequency, duration, dosage form), polypharmacy, medication duplicity, and contraindications. These errors were subcategorised into drug errors, information errors, and administrative errors (Table 1). Prescribing errors that do not fall into any of the predefined subcategories were categorised as other errors. The Prescribing Error Record Form, along with photocopied prescriptions, were delivered to the researchers for data entry, analysis, and generation of prescribing performance feedback for prescribers. As an implementation fidelity strategy, each prescription was re-evaluated for errors by the researchers [22]. Errors missed or wrongly identified during this process were promptly corrected by the researchers, and communicated to the pharmacists via email. This strategy was conducted to improve subsequent data collection by pharmacists.

**Table 1:** Subclassification of prescribing errors

Drug Error	Information Error	Administrative Error
Inappropriate dose	Duration/quantity not specified	No prescriber name/stamp
Inappropriate frequency	Dose not specified	No prescriber signature
Inappropriate duration	Frequency not specified	No date
Inappropriate dosage form	Dosage form not specified	No patient name
Polypharmacy	Strength not specified	No patient age
Medication duplication	Illegible	No patient ID
Contraindication	Abbreviation	No diagnosis
		Incorrect patient name

#### Prescribing performance feedback to prescribers

At the completion of each prescription review cycle, data on prescribing errors recorded in the Prescribing Error Record Form were analysed to generate personalised performance feedback reports for prescribers. The first batch of prescribing performance feedback was mailed to prescribers using the baseline data in May 2012. The prescribing performance feedback reports included two items. The first item was a graphical presentation of prescribing performance data—league tables (bar charts) displaying the percentage of prescribing errors at the health district, health clinic levels, as well as of individual prescribers (see Additional file 2). The specific types of prescribing error were also displayed in this item. The second item was an authorised feedback letter detailing individual prescribing error rate and prescribing performance based on a performance rating scale (see Additional File 3) [23]. This scale consisted of five levels of performance: excellent (top 10% of scale), good (top 11–30% of scale), average (middle 40% of scale), below average (bottom 11–30% of scale), and poor (bottom 10% of scale). Trends in prescribing errors were also shown in this item. Prescribing performance feedback reports were mailed to individual prescribers within two weeks of completion of each prescription review cycle. There were two exceptions—prescribing performance feedback reports for September and November were delivered to prescribers together with the reports for October and December, respectively (e.g. prescribing performance feedback reports in October contains data for September and October). The delay in the delivery of prescribing performance feedback reports was because the computer-generated dates for prescription review of the consecutive months were in close proximity; thus there was insufficient time to generate and mail reports to prescribers before the next structured prescription review cycle. To ensure that appropriate actions were taken to improve prescribing practice, a summary prescribing performance feedback report was also mailed to the district health officer of each health district, who is responsible for the administrative overview of the health district.

### Training of pharmacists

We provided training to pharmacists on prescription review, data collection, and document delivery. The training was done over three sittings, with different pharmacists according to health districts as per group allocation, to avoid treatment contamination between study groups. Pharmacists were trained on standard operating procedures, the types of prescribing errors, and the data collection process. Each pharmacist received a folder containing essential documents for the study, which included a) instructions and a flow chart on data collection and document delivery, b) operational definitions and examples of the specific types of prescribing error, c) prescriber code list containing unique identification codes for each prescriber, d) copies of the Prescribing Error Record Form, and e) researchers’ contact details. A test was administered after the training to determine the competency of pharmacists in identifying and recording prescribing errors. A set of four prescriptions with and without errors was given to each pharmacist, and each were asked to identify and record errors into the Prescribing Error Record Form. All pharmacists were required to achieve the passing mark of 85%, and those who failed received re-training until they passed the test.

### Sampling method and sample size

Using multi-stage sampling, we sampled prescriptions for review. First, we randomly selected a date in each month from a list of random numbers generated using EpiCalc 2000 [24]. This randomly selected day is the first of the five prescription collection days. Following this, we consecutively sampled prescriptions from all eligible prescribers for review.

We estimated that a minimum of 20 prescriptions per prescriber was sufficient to determine the percentage of prescribing errors each month, based on the number of prescriptions to be collected for each prescriber, the sampling unit. Assuming the percentage of errors in the average prescriber was 45% [25], the minimum errors to be detected was 15% (null hypothesis), a significance level of 5% and a power of 90%, the required sample was 19. No drop out was expected as all handwritten prescriptions were consecutively collected by pharmacists. Therefore, the number of prescriptions per prescriber to be collected was 20.

### Outcomes

The primary outcome was the percentage of prescriptions with at least one error. To evaluate the impact of the prescription review and prescriber feedback program on prescribing errors, we calculated the mean percentage error for phases 1 and 2 for each study group (see formula below). Secondary outcomes included a) the percentage of prescriptions with at least one drug error, information error, or administrative error, and b) the percentage of prescriptions with a specific type of error within each error subcategory. The denominator for calculating all percentages was the total number of prescriptions reviewed in that month.

$$ Mean\ percentage\ error=\frac{No. of\ prescriptions\ with\  at\  least\  one\  error\ in\ {phase}_x}{Total\  No. of\ prescriptions\ reviewed\ in\ {phase}_x} $$

$$ if\ x=1, prescriptions\ from\ months\ 1\  to\ 4\  are\  included $$

$$ if\ x=2, prescriptions\ from\ months\ 5\  to\ 8\  are\  included $$

### Statistical analysis

Data gathered at the end of each month was entered into SPSS version 20.0 (IBM Corp., Armonk NY) for analysis. Further statistical analysis was performed using Stata V.11 statistical software. Categorical data were presented as frequency with percentages and 95% confidence intervals. Continuous data were summarised as means with standard deviations if approximately normally distributed, or median and interquartile ranges if otherwise.

To enable the comparison of outcomes between study groups, the primary outcome, the percentage of prescriptions with error, was displayed in a statistical process control (SPC) chart [26–28]. The p-chart (p stands for proportion) was chosen because the outcome measure was binary (error versus no error) and the number of prescriptions with error and no error was known, with varying number of prescriptions (sample size) at each time point [26, 29]. The p-charts were constructed using data exported to Microsoft Excel.

### Ethical governance

This study was approved by the National Medical Research and Ethics Committee of the Ministry of Health Malaysia ((2) dlm.KKM/NIHSEC/08/0804/P12–186). In addition, permission to conduct the study was obtained from the state health director of Perak, who was in charge of public health administrative issues in the state. Confidentiality of information in the prescription was crucial, and no identifiable information on the prescription was recorded. To ensure confidentiality, names of the prescribers were not disclosed to individuals not involved in the intervention. Each prescriber was assigned a unique identification code, encrypted to preserve anonymity. Prescribers were not informed in advance about this study to prevent practice bias. Therefore, no informed consent was obtained from prescribers. However, errors identified by pharmacists during the study were communicated to the prescribers as part of their daily legal, professional, and ethical responsibilities to patients.

## Results

### Study group characteristics

The characteristics of the three study groups during the 8-month study period are summarised in Table 2. The full intervention group had a larger number of health clinics, as two of the four health districts in this group were large; this is also reflected in the volume of prescribers and prescriptions in this group.

**Table 2:** Characteristics of study groups during the study period

Characteristics	Control Group	Partial Intervention Group	Full Intervention Group
Health districts^a^, n			
Small (< 6 health clinics)	2	2	2
Large (≥ 6 health clinics)	1	1	2
Health clinics^a^, n	13	14	24
Health clinic category^a,b^, n			
Level I (> 800 patients/day)	0	0	1
Level II (501–800 patients/day)	0	0	0
Level III (301–500 patients/day)	2	0	5
Level IV (151–300 patients/day)	7	10	13
Level V (50–150 patients/day)	4	4	5
Level VI (< 50 patients/day)	0	0	0
Total prescriptions^a^, n	265,734	261,444	786,077
Average pharmacists per month^a^, n	15	14	37
Average prescribers per month, n	81	92	145
Prescriber turnover rate^c^	28.2%	34.9%	17.2%
Prescriber category, n (%)			
Family medicine specialist	3 (3)	3 (3)	2 (1)
Medical officer	60 (65)	59 (56)	107 (70)
Medical assistant	29 (32)	43 (41)	45 (29)

^a^Source: Pharmaceutical Services Division of Perak

^b^The clinics are categorised into six levels based on their daily workload, i.e. the daily average number of patients who attend the health clinic

^c^ $$ Prescriber\ turnover\ rate=\frac{No. who\  left\ during\ the\ study\ period}{\left( No. at\  the\ start\ of\ the\ study+N\mathrm{o}. at\  the\  end\  of\ the\ study\right)/2}\times 100\% $$

### Volume of prescription review

**Fig. 2:**
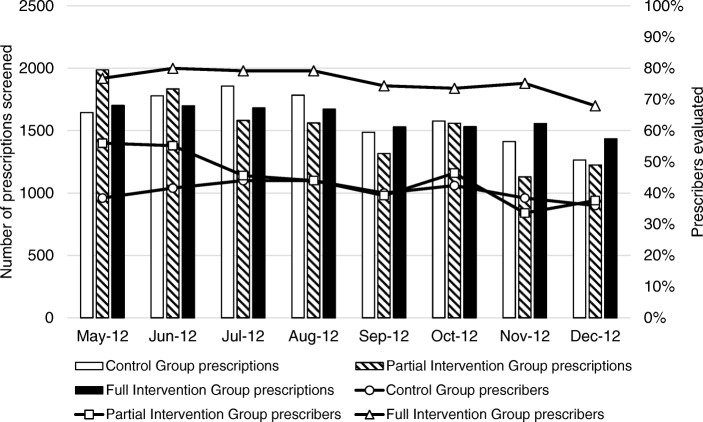
Prescriptions screened and prescribers evaluated in each study group

Figure 2 shows the volume of prescribers evaluated and prescriptions screened monthly in the three study groups. Over the 8-month study period, a total of 32,220 prescriptions were reviewed by the pharmacists. On average, more than 1000 prescriptions were reviewed and approximately 60% of prescribers were evaluated each month in each study group. We were not able to conduct prescription review for some prescribers as no prescriptions were written by them during the 5-day prescription collection window, and therefore these prescribers are not included in the analysis for that particular time point. These prescribers were on leave, did not see patients as frequently due to delegation of other work duties, or away from the clinic during the prescription review period.

### Baseline prescribing error rate

The prescribing error rate in the first month of the study represented the baseline error rate. In this month, a total of 4280 prescriptions were reviewed in all three study groups, and of these, 2055 were found to have prescribing errors. This gives an overall prescribing error rate of 48.0% (95% CI 46.5, 49.5) at baseline.

### Comparison of prescribing trend between study groups

**Fig. 3:**
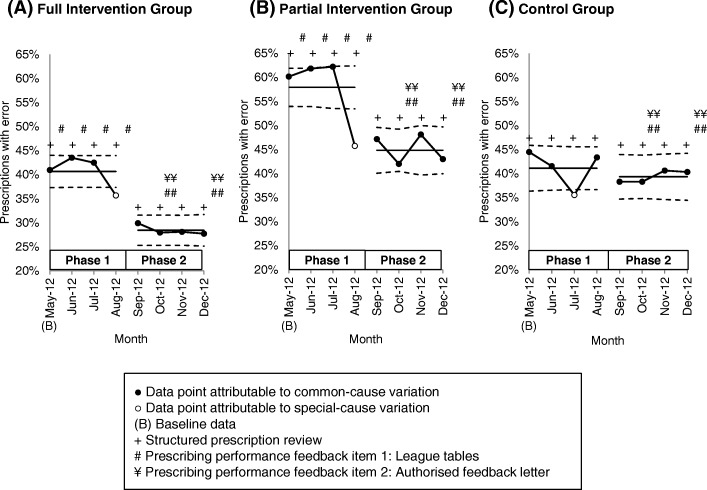
p-charts illustrating the percentage of prescriptions with error over the 8-month study period. The line with data markers represents the percentage of prescriptions with error at each time point. The central line corresponds to the process mean (average percentage of prescriptions with errors). The control limits (dotted lines) were calculated based on a normal approximation of the binomial distribution, and positioned at a distance of three standard deviations (SD) around the central line. The upper control limit was calculated by adding three times the SD to the process mean. The lower control limit was calculated by subtracting three times the SD from the process mean. The control limits for each time point was calculated based on its specific sample size (number of prescriptions), and drawn in stair-steps to reflect the changes in sample size over time. Data points within the control limits suggest common-cause variation, and data points outside the control limits suggest special-cause variation. There was a delay in the delivery of prescribing performance feedback in September and November 2012. Prescribing performance feedback reports for September 2012 and November 2012 were delivered together with the reports for October 2012 and December 2012, respectively

To compare prescribing performance between the study groups, we applied the p-chart to illustrate the impact of the prescription review and prescriber feedback program on the percentage of prescribing errors. Figure 3 compares the aggregate p-charts of the study groups over the 8-month study period. Study phases and interventions are directly annotated onto the charts.

In the full intervention group, the p-chart illustrated a positive impact of the intervention program on the prescribers (Fig. 3a). Overall, reduction in the percentage of errors occurred gradually and was sustained throughout the study period. The phase 1 p-chart indicated an average percentage of errors (central line) of 40.7% (95% CI 27.4, 29.5%). There is common-cause (random) variation in both phases 1 and 2, but less variability and more process stability was observed in the latter. There was an out-of-control episode (below the lower control limit) at the end of phase 1, suggesting an adaptation of the intervention program in the prescribing system. The phase 2 p-chart indicated an improved process with percentage of errors averaging 28.4% (95% CI 27.4, 29.5%). The full intervention program consisting of structured prescription review and a combination of league tables and authorised feedback letters likely encouraged more complete compliance, less variability, and sustained improvement in prescribing performance.

Improvement in the prescribing performance of the partial intervention group was not well sustained, and showed a seasonal pattern in the later part of the p-chart (Fig. 3b). The phase 1 p-chart indicates clearly that a less stable and inferior prescribing process was operating for the first four months of the study, with an average performance of 57.9% (95% CI 56.5, 59.3%). Similar to the full intervention group, an out-of-control episode was observed at the end of phase 1. In phase 2, the p-chart reveals a stable process with no special-cause variation, and an improved performance with percentage of errors averaging 44.8% (95% CI 43.3, 46.4%). However, the process variability in this phase is larger than that of the full intervention group, possibly due to the introduction of authorised feedback letters and the higher prescriber turnover in this group, causing changes in the prescribing system.

The prescribing performance of the control group appeared to be different from the other two study groups (Fig. 3c). As illustrated in the p-chart, although processes in both phases 1 and 2 are stable, there was no improvement in phase 2 when prescribing performance feedback was introduced into the system. The phase 1 p-chart indicated an average percentage of errors of 41.1% (95% CI 39.6, 42.6%). The single out-of-control episode in this phase could be attributed to chance (random or common-cause variation), since no prescribing performance feedback was introduced during this period (the control group received usual care in phase 1). No improvement in the process was observed in phase 2 with percentage of errors averaging 39.3% (95% CI 37.8, 40.9%).

### Subcategory of prescribing errors

**Fig. 4:**
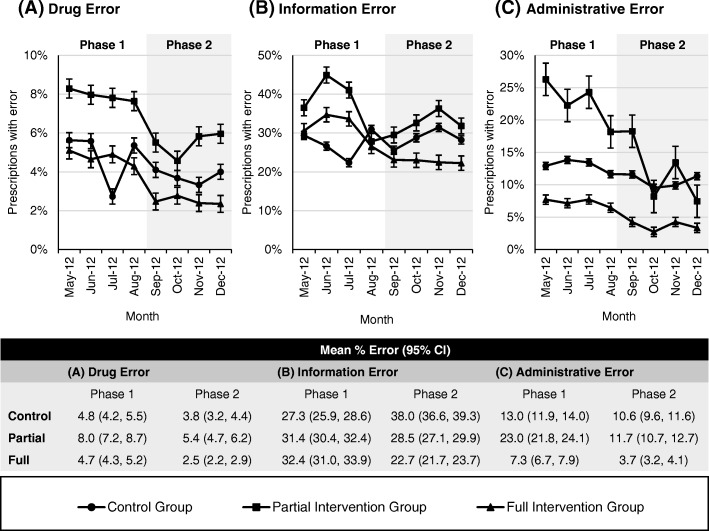
Line graphs comparing the percentage of prescriptions with error between study groups. The lines with data markers represent the percentage of prescriptions with drug error (**a**), information error (**b**), and administrative error (**c**) at each time point. Error bars indicate 95% confidence intervals

Prescribing errors identified during the structured prescription review process were subclassified into drug, information, and administrative errors. The percentage of prescriptions with these errors in each study group are illustrated in line graphs with 95% confidence intervals (see Fig. 4). Overall, there was a trend toward a reduction of all error subcategories. The reduction appeared to be more consistent in the full intervention group, regardless of error subcategory. The most frequent error subcategory committed by all study groups was information error, which consisted of incomplete prescribing information or unclear instructions on the prescription. Although there was a reduction in the percentage of prescriptions with information errors, this error subcategory appeared to be less impacted by the intervention program compared to drug and administrative errors. The mean percentage errors of each error subcategory are presented in Table 3.

**Table 3:** Types of prescribing error

	Control Group		Partial Intervention Group		Full Intervention Group	
	Phase 1 Mean % Error	Phase 2 Mean % Error	Phase 1 Mean % Error	Phase 2 Mean % Error	Phase 1 Mean % Error	Phase 2 Mean % Error
	(*n* = 4200)	(*n* = 3920)	(*n* = 5020)	(n = 3920)	(*n* = 7880)	(*n* = 7280)
Types of drug error, n (%)						
Inappropriate dose	84 (2.0)	59 (1.5)	230 (4.6)	107 (2.7)	198 (2.5)	76 (1.0)
Inappropriate frequency	59 (1.4)	51 (1.3)	72 (1.4)	37 (0.9)	48 (0.6)	35 (0.5)
Inappropriate duration	10 (0.2)	5 (0.1)	20 (0.4)	6 (0.2)	17 (0.2)	6 (0.1)
Inappropriate dosage form	8 (0.2)	2 (0.1)	13 (0.3)	12 (0.3)	49 (0.6)	6 (0.1)
Polypharmacy	36 (0.9)	18 (0.5)	75 (1.5)	26 (0.7)	64 (0.8)	31 (0.4)
Medication duplication	4 (0.1)	3 (0.1)	5 (0.1)	2 (0.1)	8 (0.1)	9 (0.1)
Contraindication	14 (0.3)	23 (0.6)	22 (0.4)	33 (0.8)	8 (0.1)	27 (0.4)
Types of information error, n (%)						
Duration/quantity not specified	241 (5.7)	178 (4.5)	444 (8.8)	250 (6.4)	507 (6.4)	339 (4.7)
Dose not specified	141 (3.4)	289 (7.4)	221 (4.4)	248 (6.3)	385 (4.9)	387 (5.3)
Frequency not specified	599 (14.3)	612 (15.6)	760 (15.1)	646 (16.5)	1319 (16.7)	872 (12.0)
Dosage form not specified	22 (0.5)	6 (0.2)	60 (1.2)	20 (0.5)	66 (0.8)	35 (0.5)
Strength not specified	41 (1.0)	45 (1.2)	168 (3.4)	32 (0.8)	107 (1.4)	68 (0.9)
Illegible	189 (4.5)	97 (2.5)	565 (11.3)	224 (5.7)	325 (4.1)	124 (1.7)
Abbreviation	201 (4.8)	221 (5.6)	468 (9.3)	288 (7.4)	510 (6.5)	246 (3.4)
Types of administrative error, n (%)						
No prescriber name/stamp	47 (1.1)	45 (1.2)	148 (3.0)	185 (4.7)	67 (0.9)	17 (0.2)
No prescriber signature	4 (0.1)	1 (0.0)	19 (0.4)	6 (0.2)	15 (0.2)	10 (0.1)
No date	8 (0.2)	17 (0.4)	14 (0.3)	8 (0.2)	19 (0.2)	8 (0.1)
No patient name	0 (0.0)	2 (0.1)	1 (0.0)	0 (0.0)	2 (0.0)	0 (0.0)
No patient age	136 (3.2)	73 (1.9)	249 (5.0)	76 (1.9)	109 (1.4)	42 (0.6)
No patient ID	8 (0.2)	11 (0.3)	11 (0.2)	4 (0.1)	14 (0.2)	6 (0.1)
No diagnosis	372 (8.9)	296 (7.6)	751 (15.0)	193 (4.9)	363 (4.6)	185 (2.5)
Wrong patient name	7 (0.2)	3 (0.1)	20 (0.4)	8 (0.2)	10 (0.1)	0 (0.0)
Other error, n (%)	76 (1.8)	45 (1.2)	240 (4.8)	90 (2.3)	162 (2.1)	112 (1.5)

### Types of prescribing error

The specific types of prescribing error within each error subcategory are summarised in Table 3. Prescribing errors that do not fall into any of the predefined error subcategories were classified as other error. Across the three study groups, the most common drug errors were inappropriate drug dose, inappropriate drug frequency, and polypharmacy. Most types of drug error appear to have decreased, but the percentage of prescriptions with contraindications have at least doubled. We hypothesised that pharmacists’ vigilance during the structured prescription review process could have led to the identification of prescriptions with antihistamines for children under 2 years old. Drug frequency not specified is the most frequent information error in prescriptions, and also the most common type of error in this study. Although overall legibility and other types of information error improved throughout the study period, increasing number of prescriptions were found with drug dose not specified. The majority of administrative errors arose from prescriptions without diagnosis, followed by prescriptions without patient age. After the initiation of the feedback interventions, violation of legal or procedural requirements of prescription writing, such as absence of prescriber’s name and signature, were reduced, although this reduction was not observed across all study groups. Prescriptions with wrong patient name, though accounting for a small proportion of the total prescriptions reviewed, are serious errors as they may cause patient harm. The numbers of such errors reduced in all groups.

## Discussion

### Principal findings

Our study explored whether monthly personalised feedback of prescribing performance to primary care prescribers using league tables and authorised feedback letters would result in reduction in prescribing error rates. Comparison between study groups, illustrated by the p-chart, demonstrated a gradual and sustained reduction in the percentage of prescribing errors in the full intervention group. Compared to the other two study groups, the process in the full intervention group was stable with less random variation. In the partial intervention group, there was also a reduction in the percentage of errors, but owing to a larger process variability, the prescribing performance was inferior to that of the full intervention group. There was no evidence of error reduction in the control group although the process was relatively stable. Overall, there is a trend toward a reduction of all error subcategories, but information errors appeared to be less impacted by the intervention program, compared to drug and administrative errors.

### Comparison with other studies

In this study, we demonstrated that mailed personalised feedback could lead to meaningful changes in prescribing behaviour of primary care prescribers. A review in 2012 reported that mailed interventions are well received by prescribers, and are able to evoke significant changes in prescribing patterns [30]. This finding is encouraging and illustrates the potential in using feedback to drive prescribing performances in primary care through benchmarking among prescribers. In addition, the feedback in our study was non-punitive and conducted in a confidential manner, an approach reported to be effective in detecting and addressing problems in several clinical settings [31]. The league tables enabled the prescribers to compare their performance with that of their peers, and likely offered them motivation to improve. The effect of authorised feedback letters acting as “report cards” appeared to strongly stimulate quality improvement [32, 33]. Furthermore, we provided feedback to individual prescribers and district health officers, which appeared to have a larger impact than feedback provided to district health officers alone [34]. Our study findings echoed previous studies that showed that audit and feedback can provide a framework for a proactive safety culture [35, 36], and direct constructive feedback to prescribers can effectively reduce errors [34, 37, 38].

We chose to compare prescribing error rates between study groups using SPC charts, as control charts would provide an intuitive technique from assessing to monitoring and improving quality of healthcare performance [39, 40]. We did not use mixed effect models to analyse the data as we recognise that only a small proportion of the variance will be explained by the explanatory variables in the model, as most of the variability in a health system cannot be measured in practice [41]. In any health system, common-cause variation is inherent, and so it becomes important to recognise special-cause variation and intervene as necessary [41]. Recent published studies have utilised the SPC chart to improve prescribing performance in an effort to reduce errors [37, 38, 42]. Other studies have used the SPC chart as a feedback tool to reduce infection rates [43], improve adherence to prescribing guidelines [44], and improve reporting of medical errors [45]. These studies have shown that the SPC chart is an effective way to communicate mistakes, continuously monitor impact of interventions, and drive sustainable improvement in patient care. Control charts have also been demonstrated as appropriate tools to aid health service decision making for health authorities and managers [46]. The advantage of SPC charts over classical statistical tests is that SPC methods integrate statistical significance tests with temporal trend of summary data, and acknowledge that unexplained variance is a reflection of random variation in a health system that is under control [41].

Our study reported an overall prescribing error rate of 48.0% at baseline, similar to the results of a study done locally in 12 conveniently selected primary care clinics [25]. In a subsequent study to reduce errors [47], the authors implemented an intervention package mainly comprising educational training, which resulted in an absolute reduction of 18.0% in the intervention group, and only 2.3% in the control group. Other studies that employed similar definitions of error as used in our study reported lower error rates. In the UK, community pharmacists reported that less than 1% of prescriptions had a prescribing error [48], and outpatient pharmacists in Norway detected errors in 2.6% of the prescriptions dispensed [49]. However, these two studies did not conduct a formal audit of prescriptions but relied on the reporting of prescribing errors by pharmacists. In both studies, the majority of prescriptions had incomplete or illegible drug information, followed by prescriptions with incomplete administrative endorsement, and a small proportion with incorrect drug information [48, 49].

### Strengths and limitations

This is the first national study to evaluate the effectiveness of providing prescribing performance feedback to primary care prescribers, with the aim of reducing prescribing errors in the primary care setting. The pragmatic design of this study, the delivery of interventions in ‘real world’ settings by staff with typical experience, and active stakeholder involvement provide the effectiveness of the interventions in everyday practice to answer questions of decision makers. Preliminary findings of this study were presented to managers in the Ministry of Health Malaysia, and plans to revise the intervention for feasible implementation is under way. We utilised a longitudinal randomised controlled study design, with repeated and regular prescription review and prescriber feedback, a methodological design strongly recommended by systematic reviews for this purpose. In addition, we incorporated implementation fidelity practices in the study design to ensure consistent intervention implementation, thus strengthening the validity of the study.

Several methodological challenges were identified in our study. The large volume of data collected each month for the generation of prescribing performance feedback reports, limited the ability of the researchers to provide timely feedback to prescribers (there was a time lag of one month between structured prescription review and the delivery of feedback in September and November). This might have interrupted the critical processes in our feedback system, thus impairing the impact of the intervention towards prescribers. We used p-charts to compare prescribing performance between study groups, as this method allows the differentiation between common-cause and special-cause variation in a system environment. However, to adequately assess changes and trends in prescribing performance on a control chart, it would be ideal to have more time points for this study (i.e. an extension of the data collection period), as it is common in practice, to evaluate prescriptions written by every prescriber over a convenient time period. In our study, baseline data from a single time point may not be adequate to monitor changes and the impact of the intervention program on the health system. However, we were unable to extend the period for collection of baseline data as there were urgent requests from managers to rectify prescribing errors in primary care. We initially planned a third phase of the study to assess the feasibility of the intervention program, and to specifically determine whether the intervention program can be implemented independently by pharmacists in the primary care clinics. Unfortunately, this phase of the study could not be conducted due to insufficient support from the pharmacists, as there was a general impression that they could not cope with the volume of work that the intervention program would entail. Extending the data collection period would require substantial resources, of which could not be supported by public sector funding. Additionally, although managers were pleased with the improvements in prescribing performance, they were in agreement that the intervention program was too labour- and resource-intensive for long-term implementation. Therefore, the data collection period for the study was limited to eight months, and the impact of the intervention program on prescribing performance was evaluated within this time period.

### Implications for clinicians and policymakers

This study shows that a high rate of prescribing errors occur routinely in primary care settings. Important measures should be taken to reduce these errors in an effort to improve and maintain prescribing safety. Routine prescriber feedback comprising league tables and a feedback letter acting as “report cards” by “warning” poorly performing prescribers and praising well-performing or compliant prescribers is effective in reducing prescribing errors. Ongoing monitoring of prescribing errors using SPC charts allows easier recognition of special-cause variation in a health system process to be targeted for improvement. Managers in primary care could adopt this intervention program along with temporal monitoring of prescribing performance as a quality improvement tool.

### Unanswered questions and future research

Future research on prescriber feedback should examine the impact of the intervention over a longer period of time to determine sustainability. Classifying prescribing errors according to clinical severity (e.g. fatal, life-threatening, serious, significant) and their potential harm to the patient should be considered for future work. The impact of an electronic prescribing system in detecting prescribing errors and allowing for corrections and computer-generated feedback, especially for administrative and information errors, should be evaluated.

## Conclusions

This study shows that the rate of prescribing errors in primary care is high, and routine feedback comprising league tables and a feedback letter could effectively reduce prescribing errors. The challenge now is to develop a feedback intervention program that is feasible and sustainable for long-term implementation.

## Additional files

Additional file 1 Prescribing Error Record Form (a standardised data collection form to record prescribing errors identified during the structured prescription review process) (DOCX 20 kb).

Additional file 2 League tables (bar charts displaying the percentage of prescribing errors for the health districts, health clinics, and of individual prescribers) (DOCX 226 kb).

Additional file 3 Authorised feedback letter (a letter signed by the state health director showing individual prescribing error rate and prescribing performance based on a performance rating scale) (DOCX 15 kb).

## Abbreviations

ADE: Adverse drug event
CI: Confidence interval
SD: Standard deviation
SPC: Statistical process control
