# Supplementary material for: The impact of a prescription review and prescriber feedback system on prescribing practices in primary care clinics: a cluster randomised trial
Source: BMC Fam Pract. 2018 Jul 19;19:120. doi: 10.1186/s12875-018-0808-4 (PMC6053727; doi:10.1186/s12875-018-0808-4)
Supplement: Supplementary file 1 — Prescribing Error Record Form (a standardised data collection form to record prescribing errors identified during the structured prescription review process) (DOCX 20 kb). [file 12875_2018_808_MOESM1_ESM.docx]

Additional file 1: Prescribing Error Record Form

**Improving Prescription Practices in Ministry of Health Primary Care Clinics**

**Prescribing Error Record Form (PERF) ^Version 2^**

**Prescribing Error Record Form (PERF) ^Version 2^**

Explanations for the type of prescribing errors in the PERF are shown below:

|  | Duration/quantity not specified - Intended period of treatment/quantity of drug to supply not stated |
| --- | --- |
|  | Dose not specified - Dose strength of drug not stated |
|  | Frequency not specified - Intended repetition of drug administration not stated |
|  | Dosage form not specified - Dosage form of drug not stated (where several exist) |
|  | Strength not specified - Prescribing “one tablet” of a drug that is available in more than one strength |
|  | Illegible - Prescription could not be read owing to illegible handwriting, and/or difficulty interpreting what was meant |
|  | Abbreviation - Writing a drug’s name using non-standard nomenclature |
|  | Inappropriate dose - Prescribing a dose which renders a particular line of treatment improper or undesirable; prescribing a drug in a dose below that recommended for the patient’s clinical condition |
|  | Inappropriate frequency - Prescribing a frequency which renders a particular line of treatment improper or undesirable |
|  | Inappropriate duration - Continuing a prescription for a longer duration than necessary; drug prescribed without an appropriate stop time when such is indicated |
|  | Inappropriate dosage form - Prescribing a dosage form that is not that intended |
|  | Polypharmacy - Prescribing two drugs of the same pharmacological category for the same indication when only one of the drugs is necessary |
|  | Medication duplication - The occurrence of repeated medication either in its generic or brand name |
|  | Contraindication - Prescribing a drug for a patient, as a result of a co-existing clinical condition (e.g. disease, pregnancy, breastfeeding), the drug is contraindicated; prescribing a drug unsuitable for the patient’s age |
|  | Other error - Prescribing error that do not fall into any of the predefined categories (e.g. drug omission, drug allergy, misspelling a drug name) |

**Prescribing Error Record Form ^version 2^ _______/_______ (Month/Year)**

**Health District: ________________ Clinic: _________________ Prescriber Code: _________________**

| **#** | Rx serial # | # of items | **No prescriber name/stamp** | **No prescriber signature** | **No date** | **No patient name** | **No patient age** | **No patient ID** | **No diagnosis** | **Wrong patient name** | **A*** | **B*** | **C*** | **D*** | **E*** | **F** | **G*** | **H*** | **I*** | **J*** | **K*** | **L** | **M** | **N** | **O*** |
| --- | --- | --- | --- | --- | --- | --- | --- | --- | --- | --- | --- | --- | --- | --- | --- | --- | --- | --- | --- | --- | --- | --- | --- | --- | --- |
|  |  |  |  |  |  |  |  |  |  |  | **Duration/quantity not specified** | **Dose not specified** | **Frequency not specified** | **Dosage form not specified** | **Strength not specified** | **Illegible** | **Abbreviation** | **Inappropriate dose** | **Inappropriate frequency** | **Inappropriate duration** | **Inappropriate dosage form** | **Polypharmacy** | **Medication duplication** | **Contraindication** | **Other error** |
| **1** |  |  |  |  |  |  |  |  |  |  |  |  |  |  |  |  |  |  |  |  |  |  |  |  |  |
| **2** |  |  |  |  |  |  |  |  |  |  |  |  |  |  |  |  |  |  |  |  |  |  |  |  |  |
| **3** |  |  |  |  |  |  |  |  |  |  |  |  |  |  |  |  |  |  |  |  |  |  |  |  |  |
| **4** |  |  |  |  |  |  |  |  |  |  |  |  |  |  |  |  |  |  |  |  |  |  |  |  |  |
| **5** |  |  |  |  |  |  |  |  |  |  |  |  |  |  |  |  |  |  |  |  |  |  |  |  |  |
| **6** |  |  |  |  |  |  |  |  |  |  |  |  |  |  |  |  |  |  |  |  |  |  |  |  |  |
| **7** |  |  |  |  |  |  |  |  |  |  |  |  |  |  |  |  |  |  |  |  |  |  |  |  |  |
| **8** |  |  |  |  |  |  |  |  |  |  |  |  |  |  |  |  |  |  |  |  |  |  |  |  |  |
| **9** |  |  |  |  |  |  |  |  |  |  |  |  |  |  |  |  |  |  |  |  |  |  |  |  |  |
| **10** |  |  |  |  |  |  |  |  |  |  |  |  |  |  |  |  |  |  |  |  |  |  |  |  |  |
| **11** |  |  |  |  |  |  |  |  |  |  |  |  |  |  |  |  |  |  |  |  |  |  |  |  |  |
| **12** |  |  |  |  |  |  |  |  |  |  |  |  |  |  |  |  |  |  |  |  |  |  |  |  |  |
| **13** |  |  |  |  |  |  |  |  |  |  |  |  |  |  |  |  |  |  |  |  |  |  |  |  |  |
| **14** |  |  |  |  |  |  |  |  |  |  |  |  |  |  |  |  |  |  |  |  |  |  |  |  |  |
| **15** |  |  |  |  |  |  |  |  |  |  |  |  |  |  |  |  |  |  |  |  |  |  |  |  |  |
| **16** |  |  |  |  |  |  |  |  |  |  |  |  |  |  |  |  |  |  |  |  |  |  |  |  |  |
| **17** |  |  |  |  |  |  |  |  |  |  |  |  |  |  |  |  |  |  |  |  |  |  |  |  |  |
| **18** |  |  |  |  |  |  |  |  |  |  |  |  |  |  |  |  |  |  |  |  |  |  |  |  |  |
| **19** |  |  |  |  |  |  |  |  |  |  |  |  |  |  |  |  |  |  |  |  |  |  |  |  |  |
| **20** |  |  |  |  |  |  |  |  |  |  |  |  |  |  |  |  |  |  |  |  |  |  |  |  |  |

Please refer to the page above for explanations of the different error types (A – O).

***Starred error categories should be recorded ‘by item’ (A-E, G-K, O).**
