# Supplementary material for: The impact of a prescription review and prescriber feedback system on prescribing practices in primary care clinics: a cluster randomised trial
Source: BMC Fam Pract. 2018 Jul 19;19:120. doi: 10.1186/s12875-018-0808-4 (PMC6053727; doi:10.1186/s12875-018-0808-4)
Supplement: Supplementary file 2 — League tables (bar charts displaying the percentage of prescribing errors for the health districts, health clinics, and of individual prescribers) (DOCX 226 kb). [file 12875_2018_808_MOESM2_ESM.docx]

Additional file 2: League tables

| **Name: [Prescriber name]** | **Prescriber code: [Prescriber code]** |
| --- | --- |

| **AUGUST 2016 Performance Review (Confidential): Total Prescribing Errors** |
| --- |

| Dear prescriber,  A study on improving prescribing practices in Ministry of Health primary care clinics is being conducted since May 2012. Your prescriber code in this study is **[prescriber code]**.  Results below are based on data collected, analysed, and interpreted by the researchers.   1. **Bar chart 1 (state level)** compares prescribing performance across health districts 2. **Bar chart 2 (district level)** compares performance across clinics within a district 3. **Bar chart 3 (clinic level)** shows performance of individual prescribers in a clinic 4. **Bar chart 4** shows the specific types of prescribing error for the state |
| --- |

1. **State level: across health districts**


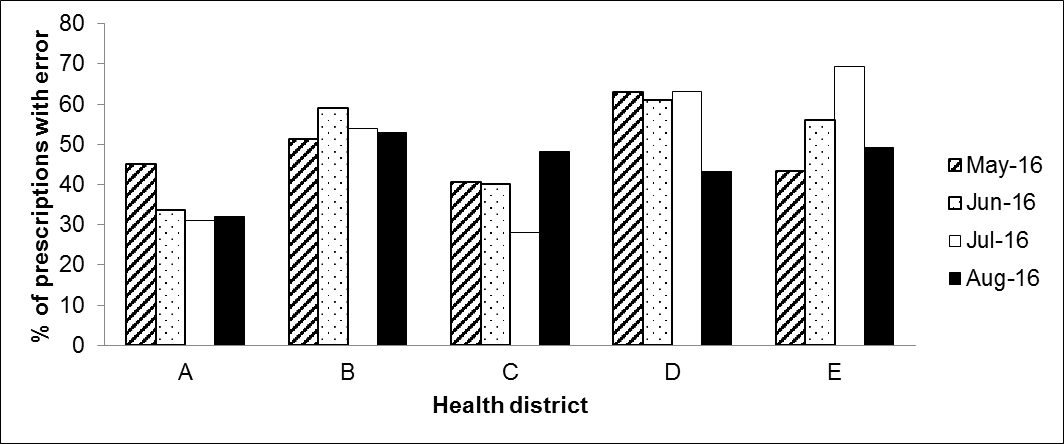


1. **District level: across clinics within a health district**


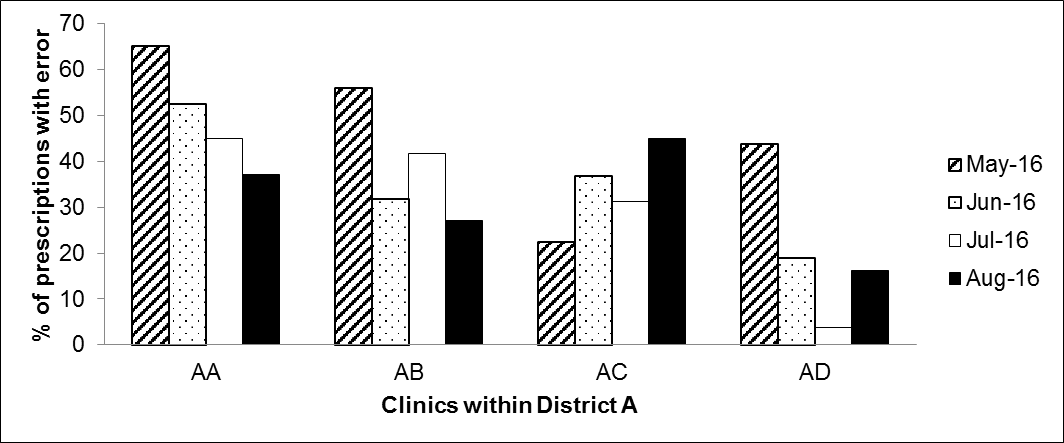


1. **Clinic level: across prescribers of clinics within a health district**


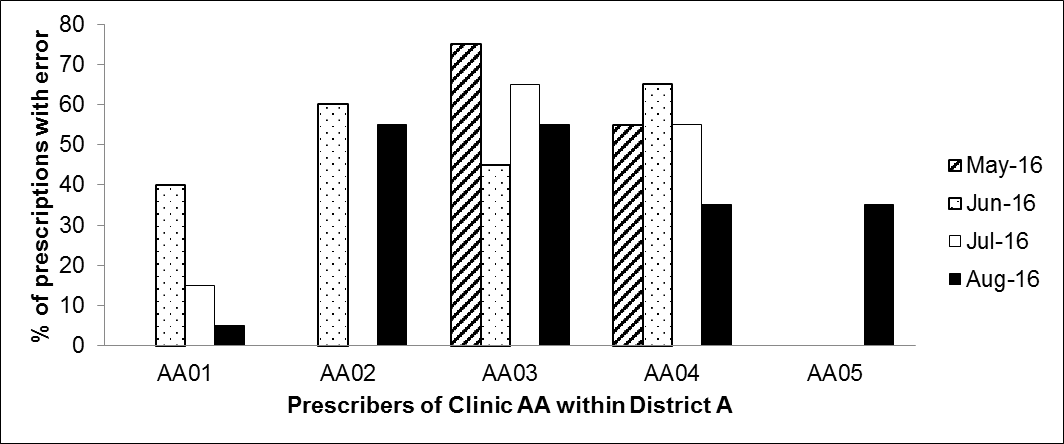


No data for AA01 in May.

No data for AA02 in May and July.

No data for AA05 in May, June, and July.


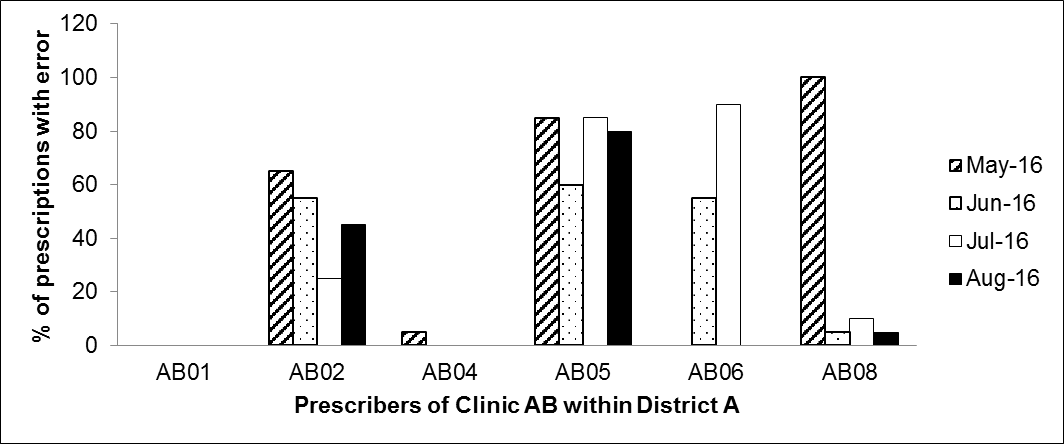


**0% error for AB04 in August.**

No data for AB01 from in May, June, July, and August.

No data for AB04 in June and July.

No data for AB06 in May and August.


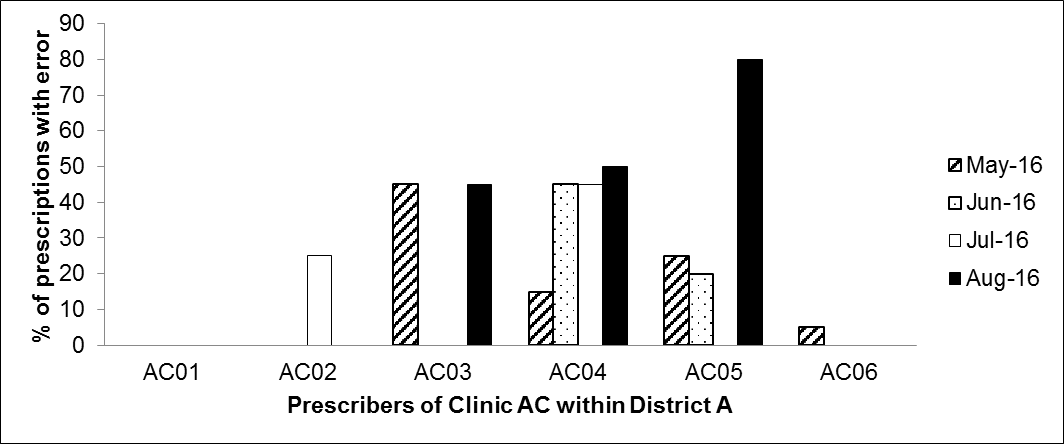


**0% error for AC01 in August.**

No data for AC01 in May, June, July.

No data for AC02 in May, June, August.

No data for AC03 in June.

No data for AC05 in July.

No data for AC06 in Jun, July, August.


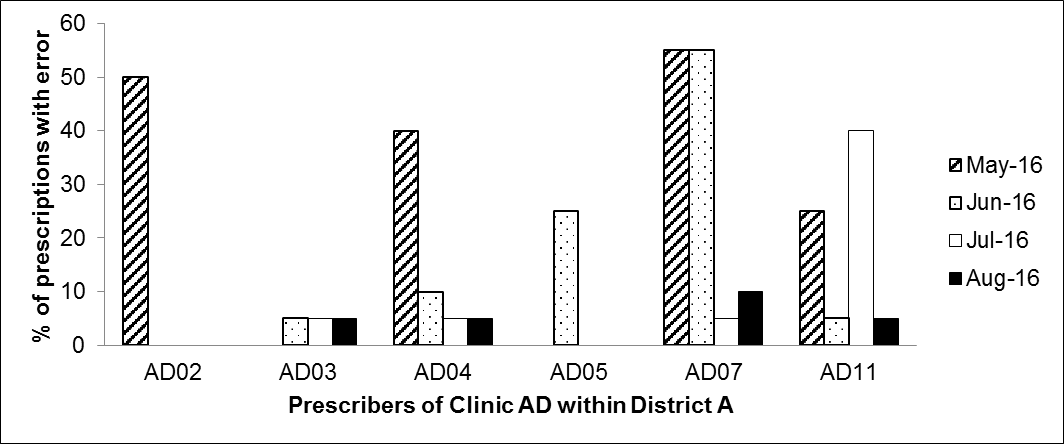


**0% error for AD02 in June and July.**

No data for AD02 in August.

No data for AD03 in May.

No data for AD05 in May, July, and August.

1. **Specific types of prescribing error for each error subcategory**


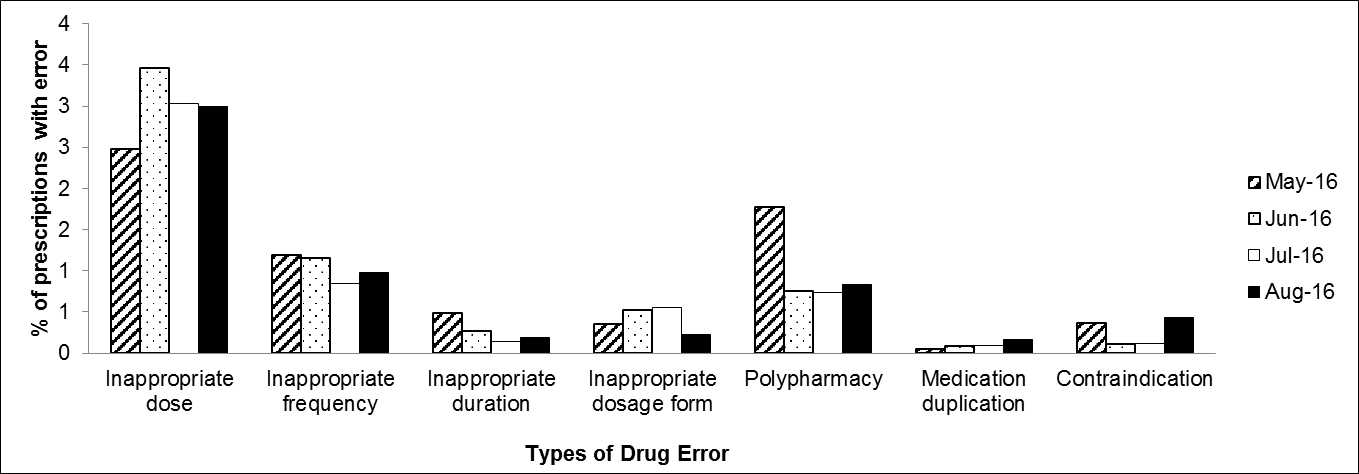


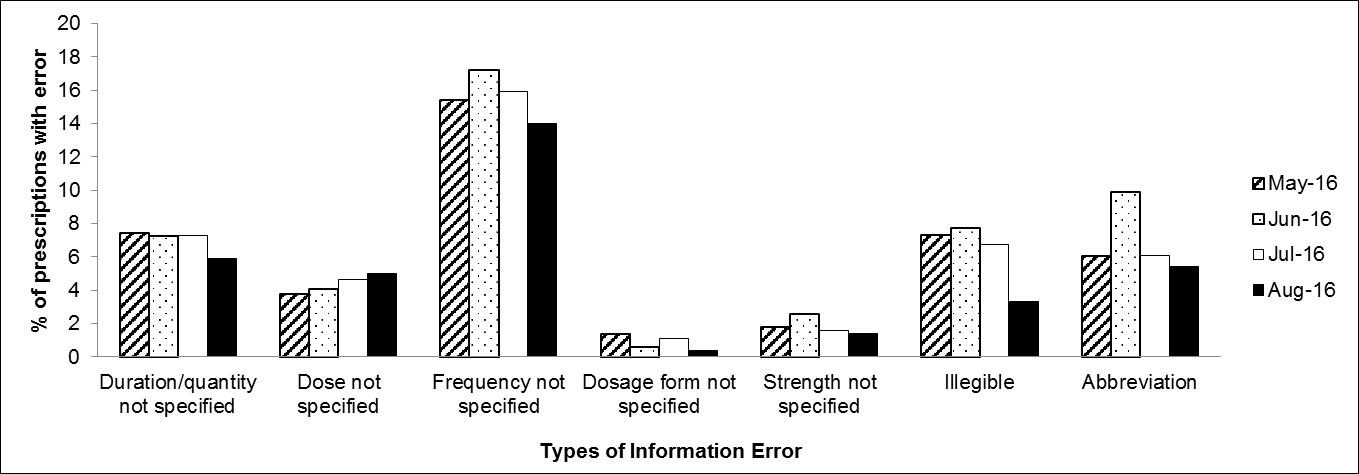


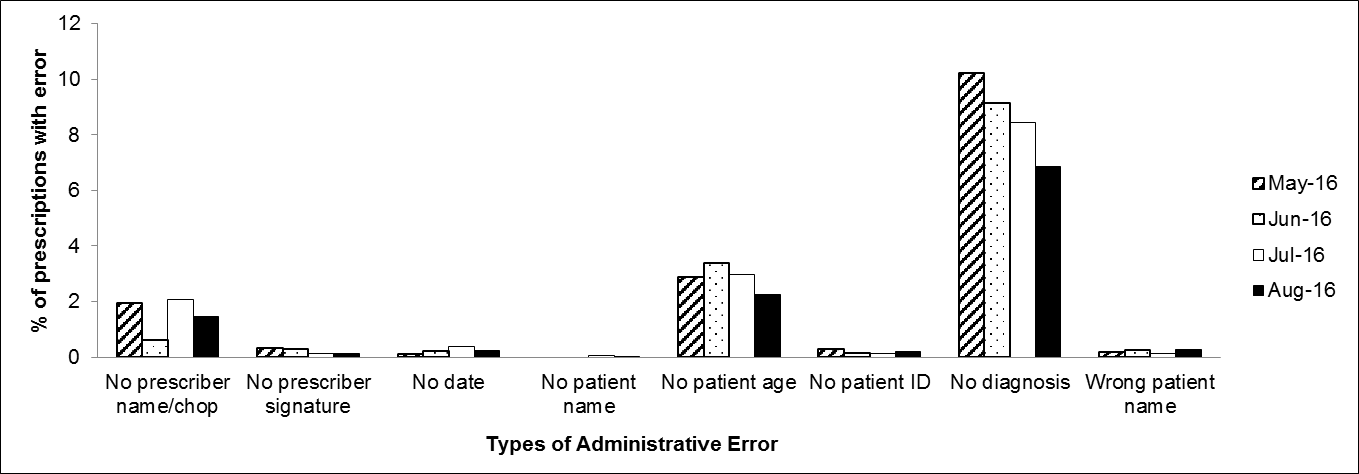


**0% error for ‘No patient name’ in May and June.**
