# Supplementary material for: The impact of a prescription review and prescriber feedback system on prescribing practices in primary care clinics: a cluster randomised trial
Source: BMC Fam Pract. 2018 Jul 19;19:120. doi: 10.1186/s12875-018-0808-4 (PMC6053727; doi:10.1186/s12875-018-0808-4)
Supplement: Supplementary file 3 — Authorised feedback letter (a letter signed by the state health director showing individual prescribing error rate and prescribing performance based on a performance rating scale) (DOCX 15 kb). [file 12875_2018_808_MOESM3_ESM.docx]

Additional file 3: Authorised feedback letter

| **Name: [Prescriber name]** | **Prescriber code: [Prescriber code]** |
| --- | --- |

| Dear prescriber,  A study on improving prescribing practices in Ministry of Health primary care clinics is being conducted since May 2012. Based on the data collected, analysed, and interpreted by the researchers, your personal prescribing error rate and performance trend are as below. |
| --- |

| State average prescribing error rate | | | | | 35% |
| --- | --- | --- | --- | --- | --- |
| Your personal prescribing error rate* | | | | | 30% |
| Drug error | 0% | Administrative error | 25% | Information error | 5% |

*Personal prescribing error rate includes drug, information, administrative, and other (not shown) error.

Based on your personal prescribing error rate above, your performance for August is:

| Excellent  (10%) |  |  |  | Well done. Your performance is excellent and your error rate is much lower (top 10%) than the state average.  Please keep up the good work & we hope you will work toward a zero error rate. |
| --- | --- | --- | --- | --- |
| Good  (20%) |  |  | 🗸 | Your performance is good and your error rate is lower than the state average (top 11-30%).  Please improve on your performance & work toward a zero error rate. |
| Average  (40%) |  |  |  | Your performance is average (middle 40%).  You are recommended to further improve your prescribing practice. |
| Below average  (20%) |  |  |  | Your performance is below average (bottom 11-30%).  You are advised to take remedial measures immediately. |
| Poor  (10%) |  |  |  | Your performance is poor (bottom 10%).  Please rectify the situation urgently. |

Based on your monthly prescribing error rates, your prescribing trend is shown below.

|  | Your performance is consistently excellent. |
| --- | --- |
|  | Your performance is improving. |
|  | Your performance is worsening. |
|  | Your performance is consistently poor. |
| 🗸 | Your performance is erratic. |

Yours sincerely,

Assistant State Health Director (Public Health)

Perak State Health Department

This is a computer-generated letter and no signature is required.
